# Gender (in)equity in global mental health research: A call to action

**DOI:** 10.1177/13634615231180376

**Published:** 2023-07-10

**Authors:** The Women in Global Mental Health Research Group, coordinated by, Kelly Rose-Clarke

**Affiliations:** 1 All group members are listed at the end of the article

In this commentary, we build on work by Gurung and colleagues which highlighted gender inequity in the global mental health research workforce in Nepal (Gurung et al., 2021). We seek to increase awareness of the under-representation of women in global mental health research and its consequences, and we call for change. By women, we refer to all people who identify as women, including trans people. The commentary is informed by conversations with women who are global mental health researchers in the Global North and South at various stages of their careers.

## The status quo

Over the last 20 years, global mental health as a research field has been criticised for biological determinism, neo-colonialism, and medical imperialism (Kirmayer & Pedersen, 2014; Mills & Fernando, 2014; Summerfield, 2013). As a community of global mental health researchers, we have responded to this critique by broadening our attention beyond the pathological to the social and structural (Lund et al., 2018), and making cultural relevance and acceptability central tenets of our work (Lund, 2020). We have committed to equitable Global North–South research collaborations, community engagement and an agenda driven by stakeholders in countries where the research is conducted (Campbell & Burgess, 2012; Patel et al., 2018). Despite our commitment to transparency and equity, we have overlooked gender disparities in our own research teams. This is illustrated by the following vignettes based on our experiences as women in global mental health research:

1. I am a project coordinator working for a non-governmental organisation in the Global South and a PhD student enrolled at a university in the Global North. The PhD funding is tied to the project coordinator role which means that I am managing a heavy workload and usually do not have time to work on my PhD during the day. I struggle to find time in the evening and on weekends because I am a daughter-in-law and, in my culture, we are expected to wake up before other family members and sleep only after finishing the household chores. Because of this I am constantly worried about falling behind. My work requires me to travel far from my family. Several times I have had to visit unfamiliar places without proper accommodation and I have felt very unsafe. I am also responsible for coordinating with local government officials and community leaders who are predominantly men AND make themselves more available and accessible to my colleagues who are men. Meetings are nearly always chaired by men and sometimes I am the only woman in attendance. Women in my culture are discouraged from speaking their minds, so in meetings I find it hard to participate in discussions. The men at the meeting usually expect me to take minutes and organise the catering. I would like to talk to my PhD supervisors about the difficulties I face, but they are both White men, not from my country, and I am afraid of losing the opportunity to do a PhD.
2. I am a mid-career researcher, a woman of African descent, based in the Global North. There is little acknowledgement within the global mental health community of the racialised dimensions of mental health work, or within academia more widely. It is clear to me that women from White European backgrounds have different career experiences and trajectories than I do, and they are more supported in their professional development. My work is often overlooked and not cited even by those who are aware of what I do. When I am invited to contribute to projects, it is only on those linked to topics around “racism” even though that is not the area of my specialisation. As a Black woman, it is hard to share how this affects me with my colleagues, and this makes for sometimes uncomfortable dialogues with peers who seem unaware of these challenges and how they affect me.
3. I am a mid-career researcher working at a university in the Global South. Because I am efficient and have strong organisational and technical skills, I am often asked to take on additional administrative tasks despite my increasing seniority. This includes organising project meetings and providing statistical support for PhD and Masters students of senior male colleagues. My early and mid-career colleagues who are men set strong boundaries around their work and don’t take on any of these additional tasks. In contrast, I find it difficult to set time aside for my own research and grant writing. I am not able to work long hours because of family responsibilities. Because my research track record is not strong and to support me, my senior colleague (who is a man) has suggested that he be the named principal investigator on the next grant application which derives from my research. My contract will come to an end with the current project and there is no bridge funding to support me until I get another grant. There is only one university funded position in Global Mental Health, which is already filled. If I am funded for a new project of less than two years duration, I will lose my sick leave, personal leave, and other benefits, and I will no longer be eligible for paid maternity leave.
4. I am an early career researcher from the Global North working on multiple projects with partners in the Global South and I am in a same-sex relationship. This can make certain work-related situations difficult to navigate, particularly if I am required to travel to countries with laws that criminalise LGBTQ+ people. It can also make forming connections with colleagues tricky as I am not sure what to say when people ask me if I have a husband or children. Although I would like to give an open answer, I am uncertain how this will impact working relationships. As a result, I don’t always feel able to build a personal rapport with colleagues, even though this is a key ingredient for long-term collaborative and trusting cross-cultural research partnerships.
5. I am a senior academic based in the Global North and working on a multi-country study in the Global South. I am pregnant and have been advised not to travel to the study sites because of the risk of catching malaria. I am not sure how I will manage to travel with a new baby: if I am to continue to breastfeed, the baby must come with me, but this means putting them at risk. I cannot afford to pay from my own pocket for someone to accompany me and help with the childcare whilst I am away. I am worried about how reducing my travel will affect the quality of my work and that I will be judged by my peers for conducting “helicopter research”. With maternity leave approaching, I am anticipating a gap in publications and funding which will delay my promotion. My institution will organise cover for my teaching but not research, so I must remain engaged with the project throughout my leave, which will cause conflict between my personal and professional life. There is a strong patriarchal culture and traditional gender divisions in the Global South countries where I work. My main collaborators in these countries are men. My Global North colleagues who are men are regularly invited to eat with my collaborators and meet their families. I have not been invited, perhaps because as a woman it is not considered honourable or appropriate.

## The gender gap in authorship

Just over a decade ago, when global mental health began to define itself as a research field, collaborations between US and UK universities and a handful of Global South organisations generated most of the research. This is reflected in the literature: a review of 467 global mental health publications between 2007 and 2016 found that five countries—South Africa, Uganda, Ethiopia, India and Nepal—were the focus of more than half the publications and most were attributed to three multi-country research consortia: PRIME (PRogramme for Improving Mental health carE), EMERALD (Emerging mental health systems in low- and middle-income countries [LMICs]) and AFFIRM (AFrica Focus on Intervention Research for Mental Health) (Misra et al., 2019). These consortia made immense contributions to global mental health research, but authorship across the related academic outputs hints at the persistent gender inequity in our field. For example, PRIME was a landmark 8-year programme, funded by the UK Government to expand coverage of mental health care in low-resource settings (Lund et al., 2012). Among the 124 PRIME publications with two or more authors published between 2012 and 2020, 59 (48%) had women lead (first) authors including junior colleagues and students. However, only 33 (27%) had a senior (last) woman author, suggesting that fewer women are leading research groups and programmes. Eleven (9%) had no women authors at all. All were authored by at least one man. We found 51 publications related to EMERALD, a programme funded through the European Commission, that focused on service system strengthening in low- and middle-income countries (Semrau et al., 2019). Of these, 23 publications (45%) had a woman lead author, 13 (25%) had a senior woman author, and 21 (41%) had no women lead or senior authors. We could not find a comprehensive list of publications from AFFIRM, one of five collaborative global mental health research “hubs” funded by the U.S. National Institute of Mental Health.

Inequities in academic authorship in global mental health are evident at a country level. For example, Gurung and colleagues conducted a scoping review to examine all mental health research about Nepal published over a 5-year period (2015–2019, inclusive of PRIME and EMERALD). They identified 337 mental health publications: 45% of first authors and 31% of senior authors were women (Gurung et al., 2021). Authorship across PRIME publications suggests women senior authors may be more likely to publish with a woman lead author: among the 33 publications with a senior woman author, 18 (55%) had a woman lead author and 15 (45%) had a man lead author. Among publications by men senior authors, 42/92 (46%) had a woman lead author and 50 (54%) had a man lead author. However, in the Nepal review, only Nepali (as opposed to non-Nepali) women senior authors were more likely to have a woman first author (Gurung et al., 2021).

Gender inequitable authorship in global mental health reflects inequities in the wider global health literature which impedes the promotion of women to more senior research roles (Table 1) (Morgan et al., 2019; Ravi et al., 2021). The proportion of women lead authors across PRIME and EMERALD and the wider global health field is higher than the proportion in the general medical literature. This may be because women represent more of the global (mental) health research workforce than the medical research workforce, or because women in global (mental) health are more likely to have lead authorship compared to women in medical research. Analysis of science, technology, engineering, mathematics and medicine (STEMM) publications including 36 million authors from more than 100 countries and over 6000 journals identified a large gap in senior authorship positions, fewer women authors in high-impact journals, and men invited to submit papers to journals at twice the rate of women (Holman et al., 2018). The gender gap in authorship can be partly explained by differences in attribution rather than scientific contribution (Ross et al., 2022). Women are less likely to be credited because their research is under-appreciated or overlooked.

**Table 1.** Representation of women authors in global mental health research publications.

Research program, initiative or publications	Number of publications reviewed	Women first authors (%)	Women senior authors (%)
PRIME [as reported in this article]	124	48	27
EMERALD [as reported in this article]	51	45	25
Mental health - Nepal only (Gurung et al., 2021)	337	45	31
Global health - Surgery (Ravi et al., 2021)	1,240	48	34
Global health - Africa only (all health fields) (Baobeid et al., 2022)	7,100	46	39
*Lancet Global Health (*Morgan et al., 2019*)*	1,323	38	30
High-impact medical journals (all health fields) (Chatterjee & Werner, 2021)	5,554	36	26
Invited commentaries (all medical fields) (Thomas et al., 2019)	34,047	27	

## The bottleneck

The under-representation of women global mental health researchers in senior authorship positions reflects the lack of senior women researchers in the field. In contrast, at a postgraduate level, women are equitably or over-represented. For example, between 2012 to 2023, 47 women (56%) versus 37 men (44%) completed the MPhil in Public Mental Health at the University of Cape Town and Stellenbosch University. At the King's College London/London School of Hygiene and Tropical Medicine Centre for Global Mental Health (CGMH), each year, for the past five years, around 90% of the student cohort (approximately 50 students) taking the global mental health MSc has been women (R. Kakuma, personal communication, December 12, 2022), but only three out of 13 CGMH professors (including honorary and emeritus professors) are women.

Across the university sector, women are more likely to be in non-tenure-track academic roles than men, with lower job satisfaction and salaries, and fewer attain senior academic positions (Rennane et al., 2022). This is borne out by the gender disparities in research funding (Jebsen et al., 2022). Wellcome, the UK's largest non-governmental funder of scientific research, found that across its 2019/20 grant portfolio (£5,150 million) fewer women than men applied for senior level awards and awards with higher value (Wellcome, 2021). Men received more than twice as much funding as women (c. £250 million compared to £115 million). Moreover, in STEMM fields, compared to men, women win fewer research grants and are awarded proportionately less of the requested sum (Jebsen et al., 2022).

Although outnumbered by men, there are examples of senior women in global mental health research who have exerted substantial influence on the field. Florence Baingana, a research psychiatrist, lobbied successfully for mental health to be included in Uganda's first health policy. She has positively shaped policy and programming in Sub-Saharan Africa whilst working for the World Bank and as Regional Advisor for Mental Health and Substance Abuse at the World Health Organization. Charlotte Hanlon is a psychiatrist, epidemiologist, and co-director of CGMH. She has been principal investigator on international consortia including PRIME, EMERALD and AFFIRM, and has built research capacity by establishing a PhD programme in mental health epidemiology at Addis Ababa University, and as the principal investigator in Ethiopia for AMARI (African Mental Health Research Initiative). Pamela Collins’ 2011 *Nature* paper (“Grand Challenges in Global Mental Health”, Collins et al., 2011) shaped priorities and funding for a decade and drove funding for mental health research in the Global South in her role as Director of the Office for Research on Disparities and Global Mental Health and the Office of Rural Mental Health Research at the U.S. National Institute for Mental Health.

These prominent figures show what is possible. However, women are rarely in senior research roles in the Global South. Although women comprised 15 of 37 individuals (41%) in the PRIME consortium, all but one of the five countries’ principal investigators were men. Out of the 51 EMERALD papers only one had a woman senior author from the Global South. Barriers to women research leaders in the Global South include lack of access to higher education, cultural expectations that women do not hold positions of authority, lack of mentorship from research leaders who are men, caring and household responsibilities, and concerns about safety in the field (Gurung et al., 2021; Owusu & Kiiru, 2014).

## Intersectionality

Under-representation of women from the Global South in global mental health research is an example of how women are further marginalised by race, ethnicity, caste, disability, and age. International and local structures, systems, culture and power all influence the way in which women are able to participate in global mental health research (Newman et al., 2017). In many countries, women researchers lack access to social welfare and protection under local labour laws. Unaffordable and inadequate childcare force some to choose between their career and their children (“You can’t have it both ways”). Our global mental health research collaborations involve organisations where cultural stereotypes and gendered practices exclude women from leadership roles, management quiz women interviewees about plans for marriage and children, and sexual harassment and bullying force women out of employment with little or no possibility of retribution. The colonial “White gaze” of global mental health overlooks the contributions and potential of Black and ethnic minority researchers (Chibanda et al., 2021; Weine et al., 2020). In extreme contexts such as Afghanistan, women's participation in public life is severely restricted. They are unable to contribute to research or if they do it is with significant risk or self-silencing through anonymity.

Women LGBTQ+ global mental health researchers are also invisible, while sexual and gender diversity remains widely criminalised. Thirty-six percent of United Nations member states (69 out of 193) criminalise consensual same-sex sexual acts, including Ethiopia and Uganda where a large proportion of global mental health research has been conducted (Pillay et al., 2022). LGBTQ+ researchers in STEMM experience more professional devaluation and career limitations than their non-LGBTQ+ peers (Cech & Waidzunas, 2021). Many feel forced out of research due to exclusionary, offensive and harassing behaviour (Powell et al., 2020).

## Why global mental health research needs to be gender equitable

In the general workforce, compelling arguments for gender equity are not new. Organisational benefits include improved innovation, productivity, job satisfaction, employee retention and decision-making (Hunt et al., 2015; Morgan Stanley, 2017). More gender-diverse companies financially outperform less diverse companies, and organisations that do not nurture women in their careers fail to make full use of and benefit from their talents (Shannon et al., 2019).

As well as these general arguments, there are specific arguments for gender equity in global mental health research. First, global mental health's commitment to a human rights-based approach is undermined by the gender-based discrimination in its research workforce (Patel et al., 2018). Second, research quality and productivity are at stake in the absence of equity. Diversity of experience and perspective are needed to solve complex, real-world problems. Underrepresented groups generate more scientific innovation than overrepresented groups but have less successful research careers (Hofstra et al., 2020). Moreover, researchers who do not feel able to disclose their gender identity or sexual orientation in the workplace publish fewer papers compared to those who have disclosed (Nelson et al., 2022). Third, how relevant are our research questions if, as global mental health researchers, we do not represent the gender composition of the populations we study? Women, who are at greater risk of depression and anxiety compared to men, and more likely to be caring for a relative with a mental health problem, ought to be a key focus for mental health intervention, yet because of the exclusion of women researchers from senior positions, men continue to set the research agenda (Riecher-Rössler, 2017). A more equitable research team will pose more relevant questions and devise more acceptable interventions (Shannon et al., 2019). Equitable representation of women is likely to direct more attention to women's priority research areas such as depressive and anxiety disorders and their relation to the impact of violence against women and girls, reproductive health and parenthood on women's mental health. The research priorities of women living in the most gender inequitable countries are at greatest risk of being sidelined. Countries ranked least gender equitable in the latest global gender gap report lack the research infrastructure or leadership to empower women to progress in global mental health research (World Economic Forum, 2022).

## Progress?

Although progress has been slow, there are signs of more gender equitable authorship in global mental health. The *Lancet* series in 2007 was a “milestone” in the emergence of global mental health as a research field (Prince et al., 2007). The series was written by the *Lancet Global Mental Health Group* of whom 29 of 39 members (74%) were men. Men were the lead and senior authors in all five of the papers in the series. In contrast, in the recent *Lancet* Commission on Global Mental Health and Sustainable Development 16 of 28 (57%) authors were men and 12 (43%) were women, although lead and senior authors were men (Patel et al., 2018).

Few global mental health programmes have explicitly focused on improving gender equity and women's leadership in research. AMARI, a grant focused on mental health research capacity building in Africa, made exceptional efforts to recruit women but faced challenges with the career pipeline, especially in Ethiopia (Chibanda et al., 2020). The Reducing Stigma Among Service Providers (RESHAPE) grant seeks to reduce stigma to improve mental health services in Nepal (Kohrt et al., 2022). Under this grant, the team is testing evidence-based approaches to gender equity among Nepali mental health researchers, including a gender equitable network of early and mid-career researchers, capacity building opportunities and mentorship schemes (Poudyal et al., 2021). The team will track the network members’ collaborations, networking, and publication outputs to measure changes in gender equity.

Across sectors, globally, progress towards gender equity slowed or even reversed during the COVID-19 pandemic (World Economic Forum, 2022). UN Women's Rapid Gender Assessment across 45 countries found that women were more likely to lose their jobs or reduce their paid work due to the pandemic compared to men (UN Women, 2021). Lockdowns, restrictions and school closures led to partnered women living with children assuming a disproportionate share of unpaid domestic work, childcare and home schooling compared to partnered men living with children (McKinsey & Company, 2020; Mooi-Reci & Risman, 2021; UN Women, 2021). Post-pandemic, some women have struggled to negotiate acceptable work patterns and more equal caring responsibilities (King's College London Global Institute of Women's Leadership & Working Families, 2021).

In research, women's productivity during the pandemic was impacted more than men's, especially among early career researchers (Andersen et al., 2020). Women wrote fewer publications and grant applications, and received fewer promotions compared to men (Fulweiler et al., 2021). In global health, women were excluded from expert and decision-making bodies with only 3.5% of 115 COVID-19 task forces around the world achieving gender parity (45–55% women) (van Daalen et al., 2020). The specific impact of the pandemic on women in global mental health research is unknown.

## The way ahead

Global mental health is an agile research field with the capacity for self-reflection and change (Bemme & Kirmayer, 2020). As a community we must now reflect on and make changes within our own research workforce. Gender inequities must be understood not just as biases (reflecting prejudicial attitudes) but as acts of discrimination (reflecting policy and individual failures). Any initiative to reduce gender inequities must work at the same time to address racism and other entwined hierarchies (Lugones, 2010). If we do not acknowledge the intersection, we risk increasing inequalities.

There must be change within research organisations (universities, non-governmental organisations), starting with transparent, formalised procedures and standards for pay and promotion decisions. These standards must expand their focus beyond grants and publications to acknowledge mentorship (teaching, training, supporting junior colleagues, team-building), administrative (coordinating, event organising) and citizenship roles (diversity and inclusion, sustainability) that disproportionately fall to women (Brommesson et al., 2022). In isolation, superficial interventions such as diversity training for men, and mentoring or capacity building schemes for women rarely work (The Prince's Responsible Business Network et al., 2020). These initiatives fail to address the underlying structural causes of gender inequity.

Organisational policies for gender equity—including caregiving allowances and support, flexibility in working locations and working hours—need to be reviewed, updated, and incentivised by the funding system. Women with flexible working arrangements (especially hybrid working or working from home) report higher career progression and job satisfaction than those without (The Global Institute for Women's Leadership, 2022). Flexible and part-time working arrangements can help some women to continue their careers and should not only be available to women in the Global North. However, such working arrangements must have clear pathways to progression and should not be misconstrued by colleagues, especially supervisors, as a lack of commitment (Jones, 2019; The Global Institute for Women's Leadership, 2022). Blind procedures for hiring and external blind reviewers for career advancement would help to mitigate conscious and unconscious bias. Organisations need to introduce and vigilantly monitor policies for women (“What gets measured gets done”). One approach is to adapt a standardised measure akin to the Global Health 50/50 “Core Variables” reporting tool (Global Health 50/50, 2022), the UN Women Gender Equality Capacity Assessment Tool (UN Women Training Centre, 2014), or the Athena SWAN (Scientific Women's Academic Network) Charter to improve gender equality in higher education and research institutions, recognising and addressing the limitations of these tools (AdvanceHE: Athena Swan Charter, 2022; Bhopal & Henderson, 2021). An organisation's gender equity rating could be an eligibility or ranking criterion for grant proposals and publication submissions.

Funding bodies and academic journals must also change. Funders must acknowledge and take responsibility for the fact that, in the current climate, global mental health research funding perpetuates and exacerbates the discrimination of women researchers, especially in the Global South. Given evidence that peer reviewers are biased towards men's research when they are not blinded to author gender, funders and journals should mainstream double blind peer and panel review (Wykes & Evans, 2020). Research funding policies that explicitly encourage and prioritise women principal investigators could help to close the gender gap among researchers, build capacity and empower women in the pipeline (NordForsk's Gender Policy is one example of this; NordForsk). Women must be equally represented on funding and recruitment panels, senior management teams, editorial boards, and peer review. Mandatory reporting of gender composition could achieve this whilst allowing for flexibility.

As a community of researchers, we need to change, and we need more data to document and drive that change. We need more research on the barriers to career progression in global mental health for women in the Global South, understanding the research ecosystems and using this to co-develop ways to address the inequities (Langhaug et al., 2020). As a community, we default to the gender binary yet many LGBTQ+ people use gender-neutral pronouns. Practising and integrating gender-neutral language, establishing LGBTQ+ staff networks with resources, implementing LGBTQ+ workplace inclusion policies and zero tolerance of sexism, homophobia and transphobia will create more supportive, diverse research environments. We also need to explore ways to support researchers in countries where LGBTQ+ people are criminalised. Critical review of research reference lists and teaching materials to ensure diversity will help to promote the work of underrepresented groups.

Residential conferences, training, meetings, and workshops are where networks and collaborations are built but are inaccessible to women who cannot travel or work outside office hours. Holding events in countries with anti-LGBTQ+ laws and practices serves to further marginalise LGBTQ+ researchers. Colleagues in the Global South may be excluded from events where they are unable to obtain the requisite visa for travel. Women researchers must be engaged in planning these events, be equally represented among speakers/facilitators, and have access to hybrid or remote participation options. This requires specific forms of support. For example, the Schizophrenia International Research Society is offering childcare services at its 2023 Annual Conference, albeit at $10 USD per hour per child (Schizophrenia International Research Society, 2023). For women who cannot or prefer not to bring their children, additional childcare costs incurred through attendance should be covered by central funding at the project, organisation, or funder level. Central funds are also needed to cover the additional childcare or travel costs associated with work on a research grant. As it stands, women cannot include these costs in the budget due to funder regulations or the need to be financially competitive with applications from men.

At a project level, efforts should be made to include remote working staff in online team meetings and social gatherings, to ensure women are not excluded from informal networking which can impact on their job satisfaction and career progression. Principles and strategies of manuscript development and authorship should be inclusive and transparent (Kohrt et al., 2014; Oliver et al., 2018). It is not enough to build the capacity of early career researchers without working to remove the bottleneck to becoming principal investigators. Senior women researchers should be equally represented in leadership roles in multi-country consortia and not limited to more junior technical roles. We need men to act as allies, confronting gender inequitable behaviour in their own teams, holding colleagues accountable, and helping women to rise through the ranks (Business in the Community, 2020).

In facing the inequity, there are opportunities for unity. Over 100 women global mental health researchers from across the globe have contributed to this commentary and founded the Women in Global Mental Health Research Group. The #MeToo movement has demonstrated the power of numbers and women's collective action against sexual violence (O’Neil et al., 2018). The 500 Queer Scientists campaign has increased the visibility of LGBTQ+ people working in STEMM (500 Queer Scientists). The Women in Global Mental Health Group is an opportunity for collective action, visibility, collaboration, and change. The Group must respond to the diverse needs and priorities of its members. To achieve this, we seek funding to bring women global mental health researchers from around the world together, virtually and in person, to define the agenda and formulate a strategy.

Addressing gender equity in global mental health research will not be easy, considering that women may themselves be perpetrators of discriminatory behaviour and that women researchers, like everyone, are part of legal, religious, and cultural ecosystems that maintain gender inequity. Nonetheless, men and women must not relent in their efforts to ensure the rights of all people are upheld. We not only need efforts to avert discrimination but also self-reflection and introspection among men and women, especially those in positions of power, to consider their own beliefs and biases and how these may be contributing to the problem. The lack of gender equity in global mental health research undermines our work, threatens our collaborations, and diminishes our relevance. For the integrity of the field, and its ability to meet its goals, we must prioritise women in global mental health research until equity is achieved.

## Members of the Women in Global Mental Health Research Group who have contributed to and signed this call to action

**Melanie Amna Abas**, MD (Res), Professor, King's College London, Institute of Psychiatry, Psychology & Neuroscience

**Binita Acharya**, BPH, Research Officer, TPO Nepal, School of Humanities and Social Sciences, Pokhara University

**Ayesha Ahmad**, PhD Reader in Global Health Humanities, St. George University London

**Shalini Ahuja**, PhD, Lecturer in Health Systems & Implementation Science, King's College London

**Katie H. Atmore**, MSc, GMH Researcher, Department of Global Health & Social Medicine, King's College London

**Sofia Astorga-Pinto**, MSc/PhDc, Health Services and Population Research Department, King's College London

**Barbara Barrett**, PhD, Reader in Health Economics, King's College London, Institute of Psychiatry, Psychology & Neuroscience

**Urvita Bhatia**, Msc, Global Challenges Research Fund Doctoral Researcher, Sangath, India and Oxford Brookes University

**Dominique Behague**, PhD, Associate Professor and Reader, Vanderbilt University & Department of Global Health & Social Medicine, King's College London

**Dörte Bemme**, PhD, Lecturer, Department of Global Health and Social Medicine, ESRC Centre for Society and Mental Health, King's College London

**Theresa Betancourt**, ScD, MA, Salem Professor in Global Practice, Boston College School of Social Work

**Mary Bitta**, Bsc, Msc, DPhil, Neurosciences department, KEMRI Wellcome Trust Research Programme

**Rochelle A. Burgess**, PhD, Associate Professor in Global Health, Institute for Global Health, University College London

**Erica Breuer**, PhD, Honorary Lecturer, College of Health, Medicine and Wellbeing, University of Newcastle, Australia

**Liana E. Chase**, PhD, Assistant Professor, Department of Anthropology, Durham University

**Cemile Ceren Sönmez**, PhD, Assistant Professor and Postdoctoral Fellow, Faculty at Koç University and Postdoc at King's College

**Enryka Christopher**, MSc, Clinical Research Specialist, 1. Trauma and Community Resilience Center, Boston Children's Hospital and Harvard Medical School; 2. Center for Global Health, University of Illinois at Chicago

**Jayati Das-Munshi**, PhD, FRCPsych, Clinical Reader, Department of Psychological Medicine, Institute of Psychiatry, Psychology & Neuroscience, King's College London

**Paola Dazzan**, PhD, Professor, Institute of Psychiatry, Psychology & Neuroscience, King's College London

**Mekdes Demissie**, PhD, Assistant Professor, College of Health and Medical Sciences, Haramaya University, Ethiopia; Centre for Innovative Drug Development and Therapeutic Studies for Africa (CDT-Africa), College of Health Science, Addis Ababa University

**Sumaiyah Docrat**, MPH (Health Economics) PhD, Health Economist & Health Systems Consultant, Independent Scholar

**Nicole D'souza**, PhD, Institute of Community and Family Psychiatry, Jewish General Hospital, McGill University

**Ngozi Enelamah**, PhD, Assistant Professor, University of New Hampshire, College of Health & Human Services, Department of Social Work

**Sara Evans-Lacko**, PhD, Associate Professorial Research Fellow, Care Policy and Evaluation Centre, London School of Economics and Political Science

**Hena Faqurudheen**, MSW, Psychotherapist, Hank Nunn Institute, India

**Gracia Fellmeth**, DPhil, Clinical Research Fellow, Nuffield Department of Population Health, University of Oxford

**Helen L. Fisher**, PhD, Professor of Developmental Psychopathology, King's College London, Institute of Psychiatry, Psychology & Neuroscience, 2. ESRC Centre for Society and Mental Health, King's College London

**Jane Fisher**, Professor of Planetary Health, Monash University

**Kimberley Goldsmith**, MPH, PhD, Professor of Medical Statistics and Complex Intervention Methodology, Department of Biostatistics & Health Informatics

**Nyaradzayi Gumbonzvanda**, Executive Director, Rozaria Memorial Trust

**Dristy Gurung**, MSc, Research Manager/PhD candidate, Transcultural Psychosocial Organization Nepal; Institute of Psychology, Psychiatry and Neuroscience, King's College London

**Rachel Greenley**, MSc, Research Fellow in Global Mental Health and Health Services Research and Policy, London School of Hygiene and Tropical Medicine

**Petra C. Gronholm**, PhD, Research Fellow, Centre for Global Mental Health, Centre for Implementation Science, Health Service and Population Research Department, Institute of Psychiatry, Psychology and Neuroscience, King's College London

**Charlotte Hanlon**, PhD, Professor, King's College London, Institute of Psychiatry, Psychology & Neuroscience, Centre for Global Mental Health; Addis Ababa University, Department of Psychiatry

**Seeromanie Harding**, PhD, Professor of Social Epidemiology, King's College London

**Weeam Hammoudeh**, PhD, Assistant Professor, Institute of Community and Public Health, Birzeit University

**Simone Honikman**, MBChB MPhil, Associate Professor and Director, Perinatal Mental Health Project, Centre for Public Mental Health, Department of Psychiatry and Mental Health, University of Cape Town

**Helen Jack**, MD, Assistant Professor, Division of General Internal Medicine, Department of Medicine, University of Washington

**Rebecca Jopling**, Research Associate, King's College London

**Hannah Maria Jennings**, PhD, Lecturer in Global Mental Health, 1. Department of Health Sciences, Univerity of York; 2. Hull York Medical School

**Jasmine Kalha**, MPhil, Research Fellow, Centre for Mental Health Law & Policy, Indian Law Society, Pune

**Malinda Kaiyo-Utete**, PhD, Mental Health Unit, Faculty of Medicine and Health Sciences, University of Zimbabwe

**Roxanne Keynejad**, PhD, NIHR Clinical Lecturer in General Adult Psychiatry, King's College London

**Hanna Kienzler**, PhD, Professor for Global Health, Department of Global Health and Social Medicine, King's College London

**Duleeka Knipe**, PhD, Senior Research Fellow, Population Health Sciences, University of Bristol

**Lily Kpobi**, PhD, Research Fellow, Regional Institute for Population Studies, University of Ghana

**Chan Lai Fong**, MD, MSc, MMedPsych, Associate Professor and Consultant Psychiatrist, UKM Medical Centre, Kuala Lumpur, Malaysia

**Joni Lee Pow**, PhD, Lecturer, Psychology Unit, Department of Behavioural Sciences, University of West Indies, St Augustine, Trinidad

**June Larrieta**, MSc, Research, Monitoring, and Evaluation Co-Lead, Ember Mental Health, SHM Foundation

**Georgia Lockwood Estrin**, PhD, Senior Lecturer, School of Psychology, University of East London

**Gergana Manolova**, MSc, Distance Learning Tutor, Department of Population Health, London School of Hygiene and Tropical Medicine

**Jenevieve Mannell**, PhD, Associate Professor, Institute for Global Health, University College London

**Lucinda Manda-Taylor**, BSoc.Sci, MA, PhD, Senior Lecturer in Applied Ethics and Health Social Science, Department of Health Systems and Policy, School of Global and Public Health, Kamuzu University of Health Sciences, Malawi

**Sarah Marks**, PhD, Director, Centre for Interdisciplinary Research on Mental Health, Birkbeck, University of London

**Kaaren Mathias**, MBChB, PhD, Advisor (Burans) and Senior Lecturer, Burans, Uttarakhand, India and Faculty of Health, University of Canterbury, New Zealand

**Dorcas Efe Mensah**, BA, Mental Health Review Tribunal, Ghana

**Leydi Moran Paucar**, BA Clinical Psychology, MPH Candidate, European Public Health Master (Europubhealth+)

**Joanna Morrison**, PhD, Senior Research Associate, Institute for Global Health, Faculty of Pop Health Sciences, University College London

**Mercilene Machisa**, MSc (Med), PhD, Specialist scientist, South African Medical Research Council; University of the Witwatersrand, School of Public Health

**Akanksha A. Marphatia**, PhD, Institute of Child Health, University College London

**Faith Martin**, PhD, Senior Lecturer, Cardiff University

**Monika Müller**, MD/PhD, Consultant Psychiatrist, Unit for Community Psychiatry, University Hospital of Psychiatry Bern, Switzerland

**Jacqueline Ndlovu**, MSc, PhD Fellow, Department of Public Health, University of Copenhagen

**Melissa Pearson**, PhD, Research Fellow, Centre for Pesticide Suicide Prevention, University of Edinburgh

**Gloria A. Pedersen**, MSc, Senior Research Associate, George Washington University

**Inge Petersen**, PhD, Professor, Centre for Rural Health, University of KwaZulu-Natal, South Africa

**Pooja Pillai**, Msc, Burans, Herbertpur Christian Hospital, Uttarakhand, India

**Mariana Pinto da Costa**, MD, MSc, PhD, Senior Lecturer, 1. Institute of Psychiatry, Psychology & Neuroscience, King's College London; 2. Institute of Biomedical Sciences Abel Salazar, University of Porto

**Praveetha Patalay**, PhD, Professor of Population Health and Wellbeing, University College London

**Anubhuti Poudyal**, MPH/PhDc, Columbia Mailman School of Public Health

**Indira Pradhan**, MA, MEd, Clinical Coordinator, Transcultural Psychosocial Organization Nepal

**Audrey Prost**, Professor of Global Health, Institute for Global Health, University College London

**Vian Rajabzadeh**, PhD, Research Associate, Imperial College London

**Meenal Rawat**, PhDc, University of Edinburgh and Burans, India

**Ursula M. Read**, PhD, Senior Research Fellow, University of Warwick

**Laoise Renwick**, BNS, PhD, Senior Lecturer, Mental Health Nursing, Division of Nursing, Midwifery and Social Work, Faculty of Biology, Medicine and Health, University of Manchester

**Tessa Roberts**, PhD, British Academy Post-doctoral Research Fellow, King's College London

**Yulia Rozanova**, PhD, Lecturer in Global Mental Health, King's College London

**Kelly Rose-Clarke**, BSc, MBPhD, Senior Lecturer in Global Mental Health, Department of Global Health & Social Medicine, King's College London

**Grace K. Ryan**, Research Fellow/PhDc, London School of Hygiene and Tropical Medicine

**Tatiana Salisbury**, PhD, Senior Lecturer in Global Mental Health, King's College London

**Manaswi Sangraula**, MPH, PhD, Assistant Director of Research, New School for Social Science Research (NSSR), Department of Psychology, New York

**Norha Vera San Juan**, PhD, Senior Research Fellow, University College London

**Poonam Sainju**, BSc, Transcultural Psychosocial Organization Nepal

**Ishrat Shahnaz**, MSc, Assistant Professor, Department of Psychology, University of Dhaka, Bangladesh

**Maya Semrau**, PhD, Senior Research Fellow in Implementation Research, Centre for Global Health Research, Brighton & Sussex Medical School

**Medhin Selamu**, MSW, PhD, Associate Postdoctoral Fellow, MHPSS and Migration officer at WHO Ethiopia, College of Health and Medical Sciences, Haramaya University, Ethiopia; Centre for Innovative Drug Development and Therapeutic Studies for Africa (CDT-Africa), Collage of Health Science, Addis Ababa University

**Seble Shewangizaw**, MPh, AMARI PhD Fellow, College of Health Science, Addis Ababa University

**Pragya Shrestha**, Clinical Coordinator/Research Fellow, Transcultural Psychosocial Organization Nepal

**Kripa Sigdel**, Psychologist/Mental Health Researcher, PhD Candidate, Department of Psychology, Tribhuvan University, Nepal

**Nikita Simpson**, PhD, Lecturer, Department of Anthropology, SOAS, University of London

**Katherine Sorsdahl**, PhD, Professor of Public Mental Health, Alan J. Flisher Centre for Public Mental Health, Department of Psychiatry & Mental Health, University of Cape Town

**Jane Brandt Sørensen**, PhD, Assistant Professor, Department of Public Health, University of Copenhagen

**Prasansa Subba**, MPhil, Project Coordinator/PhD Fellow, Transcultural Psychosocial Organization Nepal; Institute of Population Health, University of Liverpool

**Lena Skovgaard Andersen**, PhD, Assistant Professor, Director of the School of Global Health, Honorary Senior Lecturer, University of Copenhagen, University of Cape Town

**Amelia M Stanton**, PhD, Assistant Professor, Boston University

**Claire van der Westhuizen**, MBChB, PhD, Associate Professor, Alan J. Flisher Centre for Public Mental Health, Department of Psychiatry & Mental Health, University of Cape Town

**Lena Verdeli**, PhD, MSc, Associate Professor of Clinical Psychology, Clinical Psychology Program, Teachers College, Columbia University

**Marisha Wrickemsinhe**, PhD, Research Fellow, LSHTM, Department of Public Health, Environments and Society

**Samantha Willan**, PhD, Senior Researcher, South African Medical Research Council

**Angi Yoder-Maina**, Executive Director, Green String Network

**Helena Zavos**, PhD, Senior Lecturer, Department of Psychology, Institute of Psychiatry, Psychology & Neuroscience, King's College London
